# Characterization of bacterial diversity between two coastal regions with heterogeneous soil texture

**DOI:** 10.1038/s41598-022-23487-0

**Published:** 2022-11-07

**Authors:** Maryam Zakavi, Hossein Askari, Mohammad Shahrooei

**Affiliations:** 1 Department of Cellular and Molecular Biology, Faculty of Life Sciences and Biotechnology, Shahid Beheshti University, Tehran, Iran; 2 Department of Microbiology and Immunology, Clinical and Diagnostic Immunology, KU Leuven, Leuven, Belgium

**Keywords:** Biotechnology, Ecology, Evolution, Microbiology, Plant sciences

## Abstract

Studying microbial diversity and the effects of external factors on the microbiome could expand our understanding of environmental alterations. Silt and sand are mineral particles that form soil texture and even though most of the soils on earth contain a fraction of them and some other soils form almost by them, their effects on the microbiome remained to elucidate. In this study, the bacterial biodiversity of sand and silt clay soils was investigated. Furthermore, their effects on plant growth have been determined. Our data showed that biodiversity and biomass of microbiome are higher in silt-based soil. It is interesting that the *pseudomonas* genera only exist in silt-based soil while it is in the absence of sand-based soil. In contrast, *B. thuringiensis* could be found in sand-based soil while it is not found in silt texture. Our data also demonstrated that there are no significant changes in stress response between the two groups however, differential physiological changes in plants inoculated with silt and sand based bacterial isolates have been observed. This data could indicate that smaller size particles could contain more bacteria with higher biodiversity due to providing more surfaces for bacteria to grow.

## Introduction

An essential component of the Earth system is the soil sphere^1^. The soil microbiome is considered to be the main component of soil^2,3^. Soil microbiome could influence soil development, organic matter decomposition, geochemical cycles, and energy conversion^4^. In addition, it could significantly promote plant growth^5^. The biomass of the microbiome is reducing, and its biodiversity has decreased as a result of the abuse of the environment and resources, while soil bacterial diversity is a critical factor in ecosystem assessment and maintenance of ecological balance^6^. More research was concentrated on the study and preservation of soil microbial variety, the analysis of diversity traits, and the influence of variables on diversity^7,8^. Accordingly, these variables can be divided into human interventions and natural variables^9,10^. While human influences include pesticides, fertilizers, and tillage techniques, natural factors include the type of agricultural vegetation, soil type, temperature, and moisture^11–13^. Several reports suggest a relationship between soil properties and bacterial populations, although the results of individual studies vary as to the nature, extent, and direction of this relationship. According to certain experimental findings, the texture of the soil is the primary force behind microbial community organization^14,15^. Greater physical niche space and spatial isolation induced by the structural diversity of the soil environment should promote bacterial diversity^16,17^. There is evidence that soil bacteria prefer this specific texture^18–20^. Correlations between the ratio of surface area and bacterial communities have been also observed in marine sediments, suggesting that the surface area to volume ratio of mineral material may influence microbial community formation and activity^21,22^.

Regardless of the complexity of microbiome changes, there is technical complexity to determine the microbiome itself. Currently, sequencing of 16S rRNA and 18S rRNA genes provides the most accurate identification of bacteria^23^. Recently, matrix-assisted laser desorption ionization-time of flight mass spectrometry (MALDI-TOF MS) has come into focus as a potential method for the detection and diagnosis of microorganisms. Microorganisms are detected using either whole cells or cell extracts during the MALDI-TOF MS procedure. The method is sensitive, rapid, and affordable in terms of labor and other associated costs in comparison with other techniques. Microbiologists have reported that MALDI-TOF MS is used for a variety of purposes, including identification of bacteria and their strain type, epidemiological studies, and other purposes^24–26^.

Here, to identify the impact of salt and silt texture on soil, we collected samples from these two specific textures and examined bacterial diversity using MALDI-TOF MS. In addition, we determined the tolerance of bacterial isolates to abiotic stressors (salinity, alkalinity, and thermal stresses). We also investigated the effects of isolates on the growth of Maize, Canola, and Wheat and observed the growth variations of plants inoculated with the same isolates. Our data showed that silt-based soil textures are homing more bacterial types. This texture also harbors *pseudomonas* genera which is the absence in sand-based soil. Moreover, sand-based soil has a lower number of bacteria and contains *B. thuringiensis*. Our data could not identify any difference between stress responses of these two groups which might indicate no evolutionary pressure between these groups. Regardless, plant response to bacteria demonstrated that bacteria isolated from sand- and silt-based soils could change the development of plants in favor of their environmental niches. Based on this data, it could be concluded that lower size particle is correlated with higher biomass and biodiversity. This could be a result of a higher surface provided by low size particles which could provide more niches for the growth of bacteria.

## Results

### Sampling locations of soil samples and characterization of physiochemical treats

Geographical, physical, and over 10 years of synoptic data for two locations along a latitudinal gradient were provided in Table 1. The soil samples were taken from a single longitude across two different latitudes, these points were elected on a strip from semi-arid to arid regions of the north to south, and the exact longitudes with similar pH, rainfall, and temperature. Respectively, the pH spectral was 7.6 to 8.6. The temperature of locations was from 18.1 to 22.2 and the rainfall average has not differed more than 22 mm^3^. Furthermore, soil samples' texture was determined as 8, 15 and 79% of sand, silt, and clay for location A and 85, 5, and 10% of sand, silt, and clay for location B (Table 1).

**Table 1:** Selected geographic and eco-physiological characters of soil samples along with two coastal deserts A and B from 2009 to 2019.

Location description	A	B
**Geographic characters**		
Height (feet)	142	162
Latitude	37 16.47	27 3 40.87
Longitude	55 1 39.55	55 3 59.73
**Physical characters**		
Percentage of sample weight after sieving	26.34	62.66
pH	8.69	7.12
**Texture (particle composition)**		
(%) Sand	8.00	85.00
(%) Silt	79.00	5.00
(%) Clay	13.00	10.00
**Synoptic characters (10 years av.)**		
**Temperature (°C)**		
Lowest value	7.80	17.70
Max value	29.10	29.40
Ann	18.10	22.20
**Total rainfall (mm**^**3**^**)**		
Lowest value	25.30	0.00
Max value	92.20	52.50
Ann	150.20	133.25
**Wind speed (m/s)**		
Lowest value	2.25	3.11
Max value	2.30	3.80
Ann	2.70	3.50

### Isolation, characterization, and screening of the growth parameters of soil bacteria on selective media and MB medium

After collecting samples, bacterial content of these samples has been isolated via several medium. As shown in Table 2, the selective microbial media have unique and different effects on the growth and morphology of soil isolates. At a glance, it was observed that most isolates have different growth parameters and colony sizes when they are cultured in the same microbial media, however, the growth rate of isolates has shown interesting results in all microbial media. Surprisingly the number of bacteria in location A (silt) was much greater than the bacteria isolated from location B (sand). Also, Table 2, introduced a complete list of bacteria isolated from soil of locations A and B and the influences of selective microbial media and MB medium on their growth parameters. Of the 27 soil bacteria, 17 isolates belonged to location A and 10 to location B that were isolated 1 on DPM, 4 on GYM, 4 on LB, 9 on MHA, 4 on NA, 3 on NA+, and 2 on VRB. Investigation of selective microbial media showed that isolates 3, 66, 1, 31, 32, and 67 had the most growth rate (CFU/ml) after 10 h of culture, respectively. Almost all the isolates’ colonies were cream in color but there were some colonies with colorless, white, cream, yellow, grey, orange, and dark orange. Most colonies were circular and had a smooth or flat surface, with only 30 isolates showing a raised surface (Table 2). While all colonies had an irregular shape with a flat surface and were white on the MB medium. As shown in Table 2, selective microbial media were more effective than MB medium in terms of bacterial growth and morphological characterizations of isolates. At a glance, it was observed that most isolates have different growth parameters and colony sizes when they are cultured in the same microbial media, however, the growth rate of isolates has shown interesting results in all microbial media. Isolates 6, 5, 2, 1, and 132 indicated the highest growth (CFU/ml) on the MB medium, and by comparing the growth of the isolates in both MB and selective media isolates 6, 5, and 65 showed the growth rate equal or higher than other ones (MB medium/ Selective media CFU ratio).

**Table 2:** Effect of two coastal deserts on microbial growth parameters and morphological characterization of soil bacteria isolated from locations A and B.

Location	Isolate	Selective microbial media		Morphological characterization				MB medium		MB medium/selective media CFU ratio
		Medium name	CFU/ml (× 10^5^) after 10 h	Color	Colony size score	Colony shape		CFU/ml (× 10^5^) after 10 h	Colony size score	
						Top view	Side view			
A	1	MHA	12.00	Cream	3	Circular	Flat	5.00	2	0.83
	2	MHA	9.80	Orange	2	Circular	Flat	5.50	1	0.86
	3	MHA	14.00	Dark Orange	2	Circular	Flat	4.00	1	0.95
	4	MHA	8.50	Cream	7	Irregular	Flat	3.50	6	0.7
	5	MHA	9.50	Cream	2	Circular	Flat	7.50	1	1.25
	6	MHA	4.00	Colorless	2	Circular	Flat	8.00	1	1.33
	65	NA	8.00	Colorless	3	Circular	Flat	4.00	2	1.00
	66	NA	13.00	White	3	Circular	Flat	3.80	2	0.95
	67	NA	11.00	Cream	10	Irregular	Flat	3.50	6	0.92
	75	NA+	6.00	Cream	3	Irregular	Flat	3.00	2	0.77
	92	LB	5.00	Cream	3	Circular	Flat	4.00	2	0.71
	93	LB	4.50	Orange	2	Circular	Flat	2.50	1	0.19
	112	VRB	2.80	White	1	Circular	Flat	2.30	1	0.37
	122	DPM	1.00	Grey	1	Circular	Flat	2.30	1	0.44
	131	GYM	8.50	Cream	10	Irregular	Flat	4.80	9	0.53
	132	GYM	7.00	Cream	3	Circular	Flat	5.00	2	0.63
	133	GYM	9.00	Cream	5	Irregular	Flat	2.00	4	0.48
B	30	MHA	9.50	Cream	2	Circular	Raised	3.00	1	0.5
	31	MHA	12.00	Cream	6	Irregular	Flat	2.00	5	0.22
	32	MHA	11.00	Yellow	2	Circular	Flat	2.10	1	0.22
	72	NA	4.80	Cream	10	Irregular	Flat	3.00	7	0.75
	87	NA+	4.00	White	10	Irregular	Flat	2.50	9	0.39
	88	NA+	4.50	Cream	3	Circular	Flat	2.80	2	0.28
	102	LB	4.00	Cream	7	Circular	Flat	2.00	6	0.42
	103	LB	3.80	Cream	4	Circular	Flat	2.00	3	0.4
	119	VRB	3.00	White	1	Circular	Flat	3.00	1	0.36
	145	GYM	7.50	Cream	10	Irregular	Flat	3.00	8	0.43

### MALDI TOF–MS and biochemical-based identification and investigation of the impacts of abiotic stresses on bacterial growth

**Figure 1:**
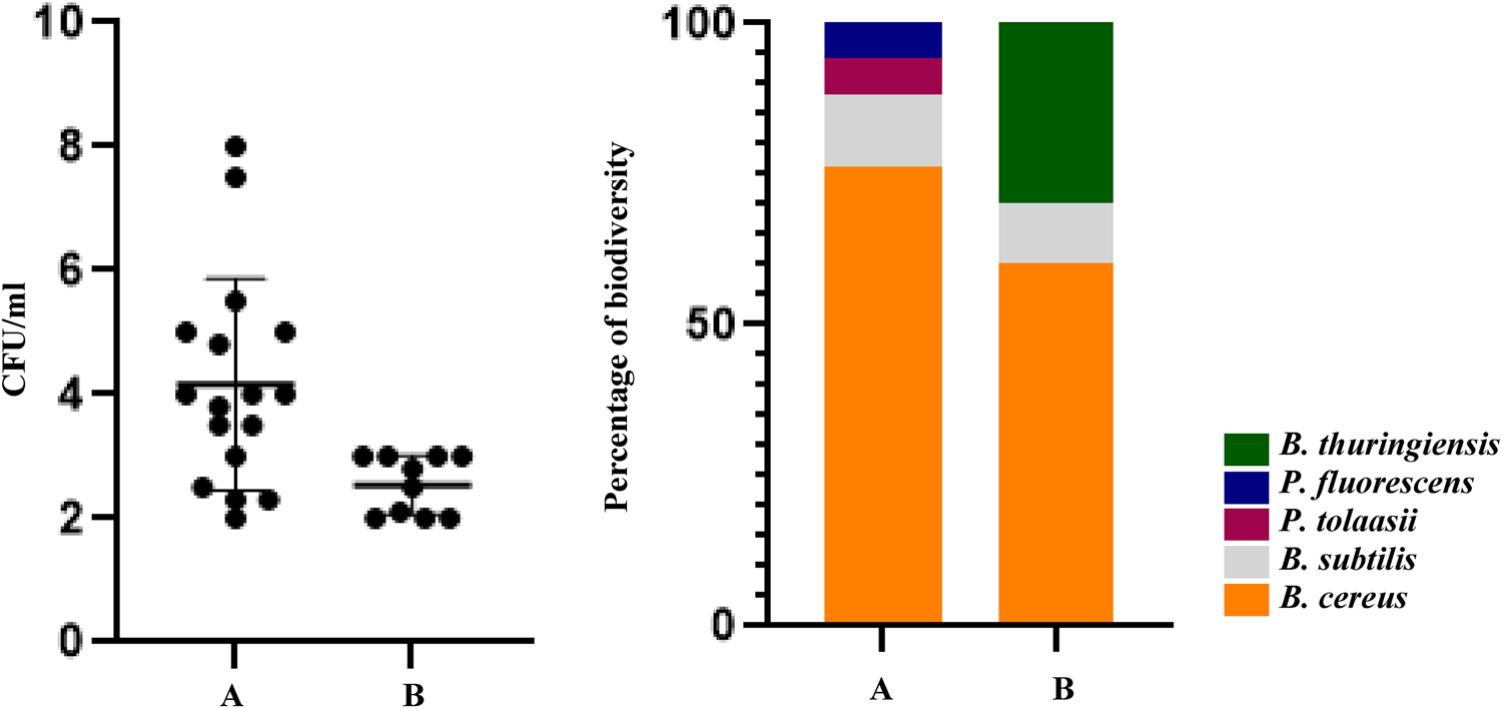
Changes in biodiversity and biomass of microbiome in response to silt and sand-based soils (**A)**. Colony counting of microbiome isolated from sampling locations (**B)**. Diversity of microbiome isolates collected from locations A and B.

Table 3, showed MALDI-TOF MS results of the 27 soil bacteria isolated from locations A and B. The obtained MALDI-TOF MS profiles were then compared to the reference spectra of the BioTyper database and their similarity was expressed by a BioTyper Log (score). In total, two different genera of Pseudomonas and Bacillus have been identified accordingly, two species belong to the *Pseudomonas* genus: *P. fluorescens* and *P. tolaasii* also three species belong to *Bacillus* genus: *B. cereus*, *B. thuringiensis*, and *B. subtilis*. The 27 soil colonies isolated from locations A and B along the transect gradient were identified by MALDI TOF MS (2 isolates (7.5%) with Log (score) ≥ 2.3; 25 isolates (92.5%) with Log (score) ≤ 2.3 and ≥ 2.0 (Table 3). Identification data showed isolates were 1 *P. fluorescens*, 1 *P. tolaasii*, 17 *B. cereus*, 4 *B. thuringiensis,* and 4 *B. subtilis*. Both locations have the same diversity of *Bacillus* genus and 3 *Bacillus* species were isolated from these regions moreover two species of *Pseudomonas* genus were isolated from location A so it could be said location A had more bacterial diversity. Interestingly, silt-based soil (location A) has higher diversity as well as microbiome biomass (Fig. 1A,B). We also test the stress response of these isolates to see the difference in tolerance in sand versus silt-based soil. Our data showed that there are no significant differences between the sand (location B) and silt soils (location A) (Table 3). The growth of all isolates under abiotic stresses was significantly reduced. Only two isolates No. 1 in cold stress and No. 145 in heat stress were able to grow close to normal condition.

**Table 3:** Investigation of bacterial diversity along with two coastal deserts by MALDI TOF and biochemical complementary tests and impacts of cold, dryness, and salinity stresses on soil bacteria isolated from locations A and B, 15 h after inoculation.

Location	Isolate	Bacterial name	MALDI-TOF MS Score	Biochemical tests				Normal condition CFU/ml (× 10^5^)	Salt stress CFU/ml (× 10^5^)	Drought stress CFU/ml (× 10^5^)	Cold stress CFU/ml (× 10^5^)	pH stress CFU/ml (× 10^5^)	Heat stress CFU/ml (× 10^5^)
				Gram staining	Catalase	KOH	Oxidase						
A	1	*B. cereus*	2.15	−	+	+	+	30.00	17.34	13.53	22.57	6.01	5.84
	2	*B. cereus*	2.08	−	+	−	+	32.00	4.44	1.63	3.87	6.32	5.54
	3	*B. cereus*	2.25	+	+	−	+	21.00	2.48	1.46	2.89	7.33	5.15
	4	*B. cereus*	2.45	+	+	+	+	25.00	3.93	1.91	2.46	9.26	6.84
	5	*P. fluorescens*	2.03	+	+	−	−	30.00	4.25	1.78	4.81	14.96	21.67
	6	*B. cereus*	2.13	−	+	−	+	30.00	4.39	2.44	4.33	12.14	9.93
	65	*B. cereus*	2.23	+	+	−	+	20.00	4.23	1.27	3.34	4.8	5.18
	66	*B. cereus*	2.04	−	+	−	+	20.00	4.28	1.56	3.57	8.74	11.6
	67	*B. cereus*	2.26	−	+	−	+	19.00	3.58	1.27	3.01	5.23	4.35
	75	*B. cereus*	2.18	−	+	−	+	19.50	5.57	1.15	3.01	6.95	7.71
	92	*B. cereus*	2.07	+	+	−	−	28.00	6.05	2.25	4.81	12.86	11.55
	93	*B. cereus*	2.11	+	+	−	+	65.00	9.17	5.07	8.38	39.02	34.77
	112	*P. tolaasii*	2.03	+	+	+	+	31.00	5.95	2.44	4.92	7.26	6.04
	122	*B. thuringiensis*	2.07	+	+	−	+	26.00	5.35	1.01	2.52	15.65	11.8
	131	*B. subtilis*	2.14	+	+	−	+	45.00	5.11	2.25	2.78	15.58	24.61
	132	*B. cereus*	2.26	+	+	−	+	39.50	4.97	1.98	2.17	19.45	21.71
	133	*B. subtilis*	2.11	+	+	−	−	21.00	3.11	1.19	1.73	13.47	18.33
B	30	*B. cereus*	2.06	-	+	−	+	30.00	5.93	1.51	2.65	13.11	7.77
	31	*B. cereus*	2.00	+	+	−	+	45.00	8.77	2.53	3.99	15.04	24.54
	32	*B. cereus*	2.17	+	+	−	+	48.00	9.21	2.5	6.52	10.12	14.24
	72	*B. cereus*	2.38	+	+	−	+	20.00	4.4	1.58	3.38	6.89	11.74
	87	*B. cereus*	2.09	+	−	−	−	32.00	4.76	2.23	3.92	17.1	10.1
	88	*B. subtilis*	2.00	+	+	+	−	50.00	7.11	3.21	5.46	31.27	21.5
	102	*B. thuringiensis*	2.06	+	+	−	+	24.00	3.29	1.29	1.84	11.71	8.23
	103	*B. thuringiensis*	2.22	+	+	−	+	25.00	4.86	1.99	3.98	7.27	6.54
	119	*B. thuringiensis*	2.05	+	+	+	+	42.00	8.22	2.33	5.01	21.02	15.81
	145	*B. subtilis*	2.01	+	+	−	−	35.00	6.44	3.55	4.03	25.86	34.34

Isolates that were undoubtedly identified to genus or species level by a valid MALDI-TOF MS score of ≥ 2.0 are shown. a and b were indicated sampling sites where the bacteria were isolated. Basal media was Muller Hinton for normal and stress conditions.

### Effects of isolates on plant growth parameters

**Figure 2:**
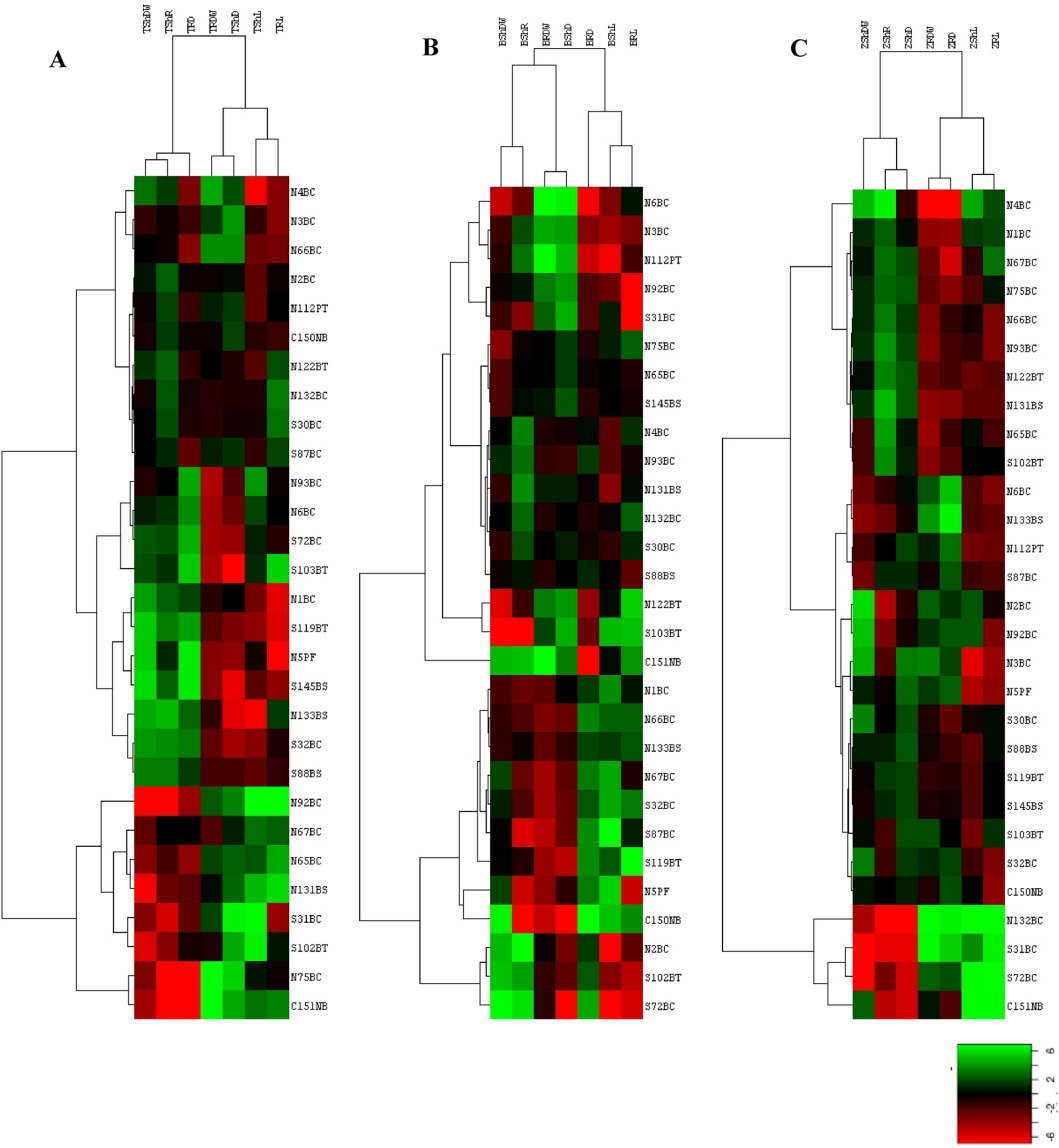
(**A–C**) Represented hierarchical cluster analysis of the effect of isolates on wheat, canola, and maize plants, respectively. Drown by CLUSTER and Treeview softwares. Hierarchical clustering was done based on Euclidian distance and the complete linkage method. Colors were indicated the type of isolates impacts on plants. Accordingly, red, green, and black colors showed positive, negative, and no-effect isolates, respectively. The horizontal axis indicates plant growth parameters: shoot density (ShD), root density (RD), root dry weight (RDW), root length (RL), shoot dry weight (ShDW), shoot length (ShL) and shoot/root weight (ShR). The vertical axis shows the assayed bacterial isolates.

**Figure 3:**
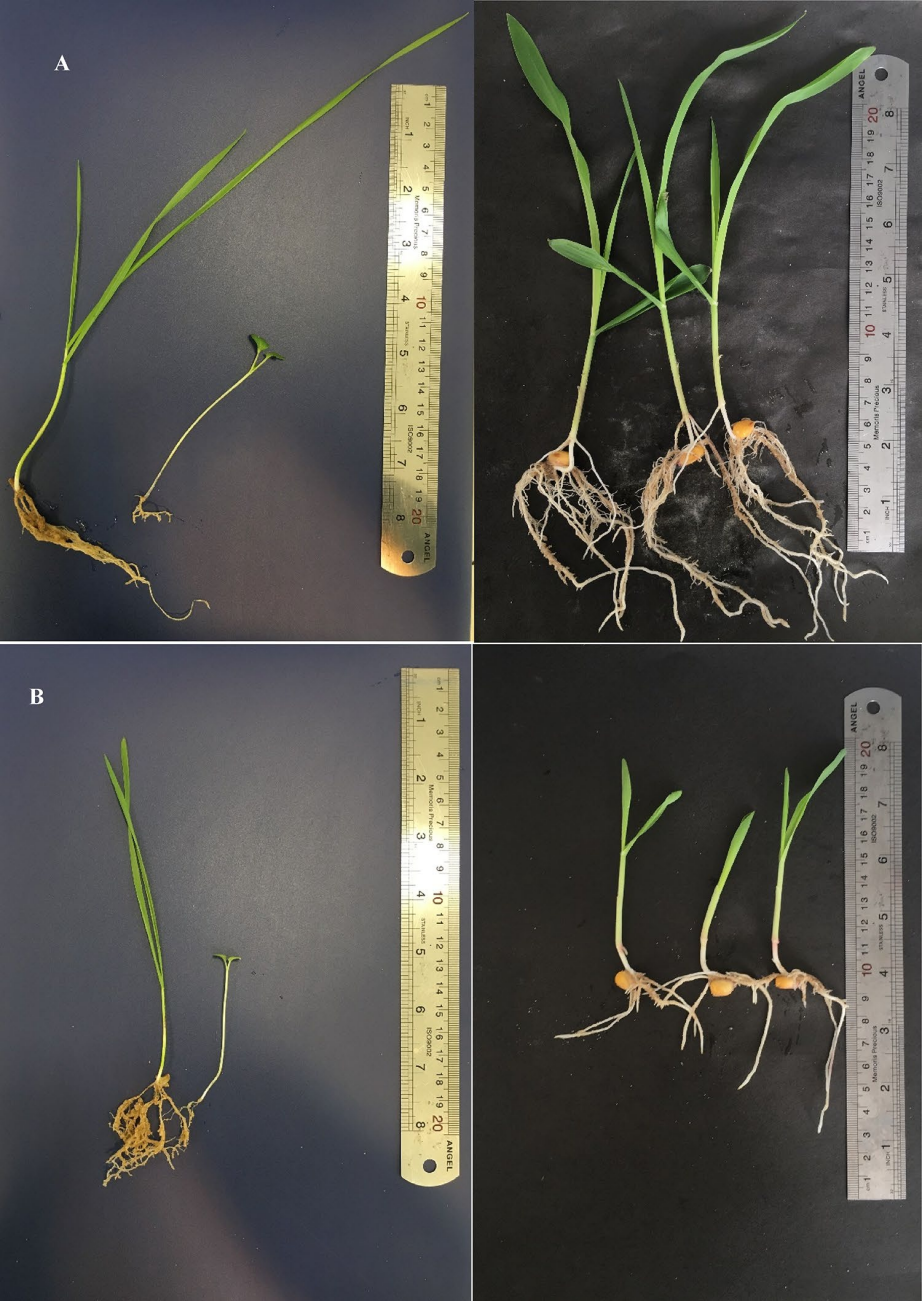
Influence of bacterial isolates on the overall growth of wheat, canola, and maize (**A**) in comparison to uninoculated control condition (**B**).

In general, all of the plant types show significant changes in response to inoculation of isolates in comparison to control condition. Plant growth parameters of wheat were strongly influenced by soil types. Results of soil bacteria effect on wheat after 21 days indicated that isolate 92 had significant impacts on shoot dry weight and shoot/root but isolate 145 caused the max reduction of shoot dry weight and root density. Furthermore isolate 93 was the most effective isolate on root weight and it was one of the most significant isolates on shoot density after isolate 103. Interestingly, isolate 133 decreased shoot/root but boosted shoot length also isolate 31 with a significant effect on root length while the reduced length of shoot, and it should be noted that isolate 5 had the best impact on root length (Table 4). By analyzing the average value of each parameter based on the isolation location of bacteria, it was found Location B was more effective on root dry weight and shoot density, while location A influenced shoot length, shoot dry weight, and root density (Table 4, Figs. 2A, 3A,B).

**Table 4:** Influence of soil isolates on growth parameters after 21 days of assessment on wheat plants.

Location	Isolate	Bacterial name	Wheat						
			Dry biomass					Length (cm)	
			mg			mg/l × 100			
			Shoot	Root	Shoot/root	Shoot density	Root density	Shoot	Root
A	1	*B. cereus*	1.96 jk	34.73 hijklm	0.22 jkl	0.69 jklmno	5.70 ghi	8.75 fgh	5.00 bcde
	2	*B. cereus*	4.02 fghij	34.91 hijklm	0.37 hijkl	1 fghijk	11.56 fghi	10.75 abcd	3.50 fghi
	3	*B. cereus*	5.36 defg	30.00 jklm	0.48 ghi	0.55 klmno	17.86 defg	11.50 ab	5.50 abc
	4	*B. cereus*	1.83 jk	11.00 op	0.18 kl	0.37 no	16.67 defgh	10.00 bcdef	3.00 hijk
	5	*P. fluorescens*	1.67 k	88.33 c	0.19 kl	1.31 efghi	1.89 i	9.00 efgh	6.75 a
	6	*B. cereus*	4.67 fgh	80.67 c	0.5 ghi	2.02 bcd	5.68 ghi	9.50 cdefg	4.00 defgh
	65	*B. cereus*	7.33 cd	26.50 lmn	0.8 ef	0.98 fghijkl	27.62 de	9.25 defg	2.75 hijk
	66	*B. cereus*	3.57 ghijk	16.43 no	0.36 hijkl	0.43 mno	21.97 def	10.00 bcdef	4.00 defgh
	67	*B. cereus*	7.50 cd	53.33 de	0.77 ef	1.48 ef	14.06 efghi	9.75 cdefg	3.75 efghi
	75	*B. cereus*	5.83 cdef	5.83 op	0.60 fgh	0.19 o	100.00 b	9.75 cdefg	3.00 hijk
	92	*B. cereus*	23.33 a	45.00 efgh	2.28 a	1.50 def	51.92 c	10.25 bcdef	3.00 hijk
	93	*B. cereus*	7.00 cde	116.00 a	0.67 efg	2.24 b	6.10 ghi	10.50 abcde	5.25 bcd
	112	*P. tolaasii*	4.50 fgh	29.17 klm	0.41 hijk	0.85 ijklmn	15.49 efghi	11.00 abc	3.50 fghi
	122	*B. thuringiensis*	3.33 ghijk	27.50 lm	0.38 hijkl	1.17 efghij	12.22 fghi	9.00 efgh	2.50 ijk
	131	*B. subtilis*	14.67 b	49.83 efg	1.28 bc	1.42 efg	29.79 d	11.50 ab	3.50 fghi
	132	*B. cereus*	5.00 efg	38.57 hijk	0.49 ghi	1.5 def	13.05 fghi	10.25 bcdef	2.75 hijk
	133	*B. subtilis*	1.83 jk	41.33 fghi	0.15 l	2.07 bc	4.45 ghi	12.00 a	2.00 jk
B	30	*B. cereus*	4.64 fgh	36.31 hijkl	0.49 ghi	1.4 efgh	12.77 fghi	9.50 cdefg	2.75 hijk
	31	*B. cereus*	8.00 c	32.00 ijklm	1.07 cd	0.47 lmno	25.00 def	7.50 h	6.75 a
	32	*B. cereus*	2.25 ijk	50.00 efg	0.23 jkl	1.67 cde	4.50 ghi	10.00 bcdef	3.00 hijk
	72	*B. cereus*	3.50 ghijk	84.75 c	0.36 hijkl	2.12 bc	4.08 ghi	9.50 cdefg	4.00 defgh
	87	*B. cereus*	4.17 fghi	25.00 mn	0.45 ghij	0.9 ghijklm	16.67 defgh	9.25 defg	2.75 hijk
	88	*B. subtilis*	2.50 hijk	38.33 hijk	0.28 ijkl	1.19 efghij	6.52 ghi	9.00 efgh	3.25 ghij
	102	*B. thuringiensis*	13.93 b	62.74 d	1.33 b	1.05 fghijk	22.23 def	10.5 abcde	6.00 ab
	103	*B. thuringiensis*	3.93 fghij	104.40 b	0.39 hijkl	5.22 a	3.71 hi	10.25 bcdef	2.00 jk
	119	*B. thuringiensis*	1.55 k	51.07 ef	0.16 l	1.09 fghij	3.03 hi	9.75 cdefg	4.75 bcdef
	145	*B. subtilis*	1.55 k	82.26 c	0.16 l	2.12 bc	1.89 i	9.75 cdefg	4.00 defgh
	150	Control +	5.00 efg	40.00 ghij	0.45 ghij	0.89 hijklmn	12.88 fghi	11.00 abc	4.50 cdefg
	151	Control -	7.00 cde	4.00 p	0.85 de	0.23 o	175.00 a	8.25 gh	1.75 k

LSD p = 0.01 value for the parameters on wheat, respectively: 0.003243491, 0.05366804, 0.05586241, 0.1312794, 3.136652, 2.177158, 4.041771; A and B were indicated sampling locations.

Plant growth parameters of Canola were also strongly influenced by the size of the particle. Results of the effect of soil bacteria on canola after 21 days indicate that 103 had significant impacts on shoot dry weight and shoot/root however isolate 72 caused reducing these parameters. However, no isolate was more appropriate for root dry weight, shoot density, and root density than the control. Isolate 131 showed the max shoot length but isolate 32 caused the lowest length of the shoot. Also, isolate 31 indicated the best root length (Table 5). Hierarchical cluster analysis of effects of isolates on canola showed that location B was more effective on root dry weight, shoot/root, and shoot density, while location A had a good influence on shoot length, root length, root density and shoot dry weight (Table 5, Figs. 2B, 3A,B).

**Table 5:** Influence of soil isolates on growth parameters after 21 days of assessment on canola plants.

Location	Isolate	Bacterial name	Canola						
			Dry biomass					Length (cm)	
			mg			mg/l × 100			
			Shoot	Root	Shoot/root	Shoot density	Root density	Shoot	Root
A	1	*B. cereus*	22.68 cdef	315.98 def	0.94 bc	2.5 efgh	7.20 b	24.25 def	12.75 cde
	2	*B. cereus*	9.29 l	212.74 hijklm	0.34 lm	2.18 ghij	4.36 b	27.25 bc	9.75 hijkl
	3	*B. cereus*	17.02 hijk	125.36 nopq	0.65 fghij	1.06 klm	13.58 b	26.25 cd	12.00 defg
	4	*B. cereus*	16.43 ijk	226.43 ghijk	0.63 hij	2.21 fghij	7.26 b	26.25 cd	10.25 fghijk
	5	*P. fluorescens*	15.71 jk	357.14 cd	0.79 cdefgh	2.35 fghi	4.40 b	20.00 i	15.25 ab
	6	*B. cereus*	23.93 cde	96.07 opq	0.87 cde	0.87 m	25.02 b	27.50 abc	11.00 efghij
	65	*B. cereus*	21.34 defg	232.14 ghijk	0.81 cdefg	1.83 hij	9.22 b	26.25 cd	12.75 cde
	66	*B. cereus*	25.00 bc	468.57 b	0.85 cde	3.68 c	5.34 b	29.50 ab	12.75 cde
	67	*B. cereus*	15.36 jk	339.40 de	0.75 defghi	2.90 def	4.58 b	20.50 hi	11.75 defgh
	75	*B. cereus*	24.64 bcd	231.43 ghijk	0.93 bcd	2.01 hij	10.70 b	26.50 cd	11.50 defghi
	92	*B. cereus*	15.71 jk	149.29 mnop	0.63 ghij	1.03 klm	10.53 b	25.00 cdef	14.50 abc
	93	*B. cereus*	15.83 jk	272.50 fgh	0.58 ijk	2.37 efghi	5.82 b	27.50 abc	11.50 defghi
	112	*P. tolaasii*	14.73 k	88.93 pq	0.58 ijk	0.89 lm	16.55 b	25.75 cde	10.00 ghijk
	122	*B. thuringiensis*	28.00 b	156.00 lmno	1.07 b	1.58 jkl	17.95 b	26.25 cd	10.00 ghijk
	131	*B. subtilis*	19.46 fghi	216.34 hijkl	0.65 fghij	1.89 hij	8.98 b	30.00 a	11.50 defghi
	132	*B. cereus*	15.00 k	179.17 jklmn	0.70 efghi	2.01 hij	8.37 b	21.50 ghi	9.00 jkl
	133	*B. subtilis*	21.67 cdefg	321.07 def	0.82 cdef	2.79 defg	6.75 b	26.50 cd	11.50 defghi
B	30	*B. cereus*	18.57 ghij	203.57 ijklm	0.73 efghi	1.90 hij	9.12 b	25.50 cdef	10.75 efghij
	31	*B. cereus*	18.57 ghij	167.14 klmn	0.81 cdefgh	1.04 klm	11.11 b	23.00 fgh	16.25 a
	32	*B. cereus*	15.67 jk	290.83 efg	0.80 cdefgh	3.05 cde	5.38 b	19.50 i	9.50 ijkl
	72	*B. cereus*	4.29 m	260.00 fghi	0.18 m	2.89 def	1.65 b	24.00 defg	9.00 jkl
	87	*B. cereus*	19.40 fghi	414.40 bc	0.94 bc	3.38 cd	4.67 b	20.50 hi	12.25 def
	88	*B. subtilis*	18.57 ghij	272.26 fgh	0.70 efghi	2.02 hij	6.82 b	26.50 cd	13.50 bcd
	102	*B. thuringiensis*	9.29 l	235.83 ghij	0.40 kl	2.10 ghij	3.93 b	23.50 efg	11.25 efghi
	103	*B. thuringiensis*	41.67 a	225.24 ghijk	1.52 a	1.76 ij	18.56 b	27.50 abc	12.75 cde
	119	*B. thuringiensis*	20.71 efg	430.54 b	0.77 cdefgh	4.78 b	4.82 b	27.00 bc	9.00 jkl
	145	*B. subtilis*	20.00 fgh	197.50 ijklm	0.80 cdefgh	1.65 jk	10.13 b	25.00 cdef	12.00 defg
	150	Control +	10.12 l	668.45 a	0.50 jkl	7.86 a	1.50 b	20.50 hi	8.50 kl
	151	Control -	10.18 l	78.75 q	0.49 jkl	1.05 klm	54.29 a	20.50 hi	7.75 l

LSD p = 0.01 value for the parameters on canola, respectively: 0.001935741, 0.009622568, 0.01059692, 0.08514634, 1.476113, 1.482904, 2.118454; A and B were indicated sampling locations.

Maize growth patterns show substantial changes due to isolates inoculation. Results of the effect of soil bacteria on Maize after 14 days indicate that 132 and 72 had significant impacts on shoot dry weight, respectively. Furthermore, isolate 132 also had the best effects on shoot/root and shoot density. Isolate 4 and 65 had the most effective isolates on root dry weight and root length parameters, respectively, although isolate 2 had a bad impact on root dry weight and root length. Isolate 92 decreased shoot length nevertheless Isolate 87, in addition to having significant effects on root length, also had the best effect on shoot length (Table 6). By examining the hierarchical clustering based on the isolation and their effect on growth parameters, it was found that location B was more effective on shoot dry weight and shoot density, while location A affected the rest of the parameters (Table 6, Figs. 2C, 3A,B).

**Table 6:** Influence of soil isolates on growth parameters after 21 days of assessment on maize.

Location	Isolate	Bacterial name	Maize						
			Dry biomass					Length (cm)	
			mg			mg/l × 100			
			Shoot	Root	Shoot/root	Shoot density	Root density	Shoot	Root
A	1	*B. cereus*	31.78 b	658.28 bcdefg	53.29 b	0.27 b	8.22 bc	13.00 fghi	8.00 hijkl
	2	*B. cereus*	13.48 b	167.25 g	127.12 b	0.19 b	2.76 c	7.25 jk	5.75 lm
	3	*B. cereus*	16.77 b	207.01 fg	101.15 b	0.11 b	2.65 c	16.00 abcdef	8.25 ghijkl
	4	*B. cereus*	24.55 b	1210.85 a	23.84 b	0.24 b	17.4 a	10.00 ij	7.25 jkl
	5	*P. fluorescens*	26.68 b	310.03 efg	82.90 b	0.15 b	3.02 c	17.25 abcde	10.00 bcdefghij
	6	*B. cereus*	43.93 b	358.90 defg	129.37 b	0.24 b	2.88 c	18.00 abcde	12.25 abcd
	65	*B. cereus*	51.93 b	1016.53 ab	51.24 b	0.29 b	7.72 bc	18.25 abcd	13.50 a
	66	*B. cereus*	31.63 b	671.98 bcdefg	46.70 b	0.21 b	5.78 bc	15.00 cdefg	11.75 abcdef
	67	*B. cereus*	34.13 b	658.66 bcdefg	52.52 b	0.20 b	9.68 b	16.50 abcdef	7.00 jklm
	75	*B. cereus*	33.30 b	656.63 bcdefg	55.72 b	0.19 b	7.61 bc	17.50 abcde	8.50 fghijkl
	92	*B. cereus*	13.75 b	182.58 g	90.49 b	0.19 b	2.46 c	7.25 jk	7.00 jklm
	93	*B. cereus*	35.55 b	837.68 abcde	47.69 b	0.21 b	6.71 bc	17.75 abcde	13.00 ab
	112	*P. tolaasii*	41.75 b	445.83 cdefg	98.47 b	0.21 b	3.7 bc	19.50 ab	12.00 abcde
	122	*B. thuringiensis*	37.08 b	741.13 abcdef	50.34 b	0.19 b	6.39 bc	19.50 ab	11.50 abcdefg
	131	*B. subtilis*	35.38 b	929.08 abc	38.50 b	0.18 b	7.88 bc	19.25 ab	11.75 abcdef
	132	*B. cereus*	201.63 a	455.85 cdefg	8202.83 a	1.32 a	4.59 bc	17.50 abcde	9.00 defghijkl
	133	*B. subtilis*	45.18 b	273.18 fg	170.98 b	0.26 b	2.44 c	17.50 abcde	11.25 abcdefgh
B	30	*B. cereus*	18.69 b	312.59 efg	61.03 b	0.17 b	4.9 bc	10.75 hij	6.25 klm
	31	*B. cereus*	77.72 b	246.00 fg	436.21 b	0.57 ab	3.55 bc	14.25 defgh	6.75 jklm
	32	*B. cereus*	18.28 b	241.80 fg	80.46 b	0.16 b	2.89 c	11.00 ghij	8.00 hijkl
	72	*B. cereus*	127.43 ab	468.13 cdefg	251.99 b	0.78 ab	7.06 bc	14.50 cdefgh	7.00 jklm
	87	*B. cereus*	51.88 b	592.08 bcdefg	88.04 b	0.26 b	4.64 bc	20.00 a	12.75 abc
	88	*B. subtilis*	29.00 b	417.50 cdefg	69.71 b	0.19 b	5.49 bc	15.75 bcdef	7.75 ijkl
	102	*B. thuringiensis*	49.48 b	890.32 abcd	55.65 b	0.27 b	8.09 bc	18.25 abcd	11.00 abcdefghi
	103	*B. thuringiensis*	29.59 b	301.69 fg	115.93 b	0.18 b	4.31 bc	16.25 abcdef	7.00 jklm
	119	*B. thuringiensis*	39.12 b	615.57 bcdefg	76.49 b	0.21 b	6.08 bc	18.50 abc	9.50 cdefghijk
	145	*B. subtilis*	35.88 b	486.64 bcdefg	73.22 b	0.21 b	5.31 bc	17.00 abcdef	8.75 efghijkl
	150	Control +	31.50 b	451.28 cdefg	83.86 b	0.23 b	3.88 bc	14.00 efghi	11.75 abcdef
	151	Control -	21.93 b	304.23 efg	184.34 b	0.30 b	4.76 bc	4.75 k	3.75 m

LSD p = 0.01 value for the parameters on maize, respectively: 0.1151398, 0.5349834, 4.409074, 0.007713552, 0.06318265, 4.12234, 3.276811; A and B were indicated sampling locations.

Concerning plant–microbe interactions, it should be noted that the effect of bacteria on plant growth is limited to the plant species or plant-specific. Hierarchical cluster analysis of plant–microbe interactions of three plants maize, canola, and wheat demonstrated all thee plants growth parameters for each plant were evaluated by several isolates and surveyed isolates created unique developmental effects in different plants (Fig. 2).

**Figure 4:**
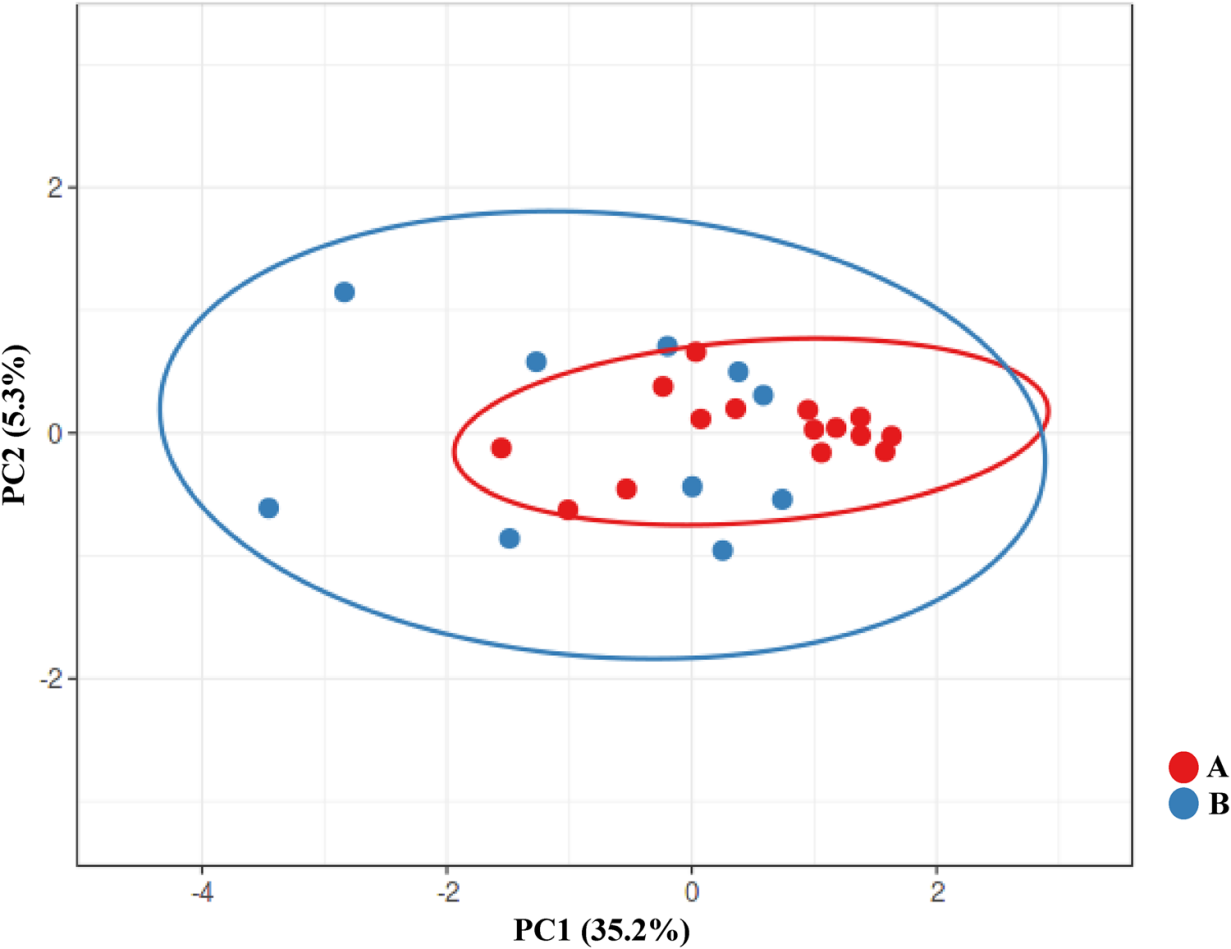
PCA analysis of all growth parameters of wheat, maize, and canola treated by isolates collected from locations A (silt) and B (sand).

PCA analysis of Plant growth parameters of Wheat, Canola, and Maize inoculated by bacteria isolated from sand (location B) and silt (location A) soils demonstrated that silt-based soil in location A has lower variation among themselves which might indicate lower evolutionary pressure (Fig. 4).

## Discussion

Evidence from several indicates that there is a connection between soil texture and microbial populations, although each study's findings on the nature, degree, and direction of this connection vary^30^. According to specific experimental findings, texture is the primary force behind the organization of microbial communities. For instance, an experiment revealed that altering the particle size distribution had a bigger effect on the organization of the microbial community than did compaction or changing the pH^15^. Previous research has discovered an association between soil textural heterogeneity as characterized by a single fractal model and microbial biomass^31^. Due partly to the considerable impact of pH on microbial diversity in natural environments, most field investigations have discovered a significant but lessened impact of texture upon microbial communities. As there is no consistent correlation between soil particle size heterogeneity and texture size classes across landscapes, these seemingly contradictory results might still be explained by a positive link between bacterial diversity and soil particle size heterogeneity^31–36^.

It has been suggested that a higher particle size could result in higher bacterial biomass^30^. Although this assumption might be right, the higher surface could give more niche for bacteria to adhere to. In this perspective lowering the size of particle in soils might result in higher biomass and diversity which might be an explanation for why silt-based soil have higher biomass and slightly higher biodiversity. Also, changes in soil texture result in biodiversity and biomass of soil which in turn affect the ecosystem and plants either. As it has been proved, microbes can evolve on short time scales, therefore shifting plant–microbe interactions quickly, boosting plant growth, and altering how we scale ecosystem processes up to longer periods^37,38^. Regarding the effects of bacteria on agriculture, it is previously known that some microbes have amazing impacts on plant performance, and changing their biodiversity could have a substantial impact on the environment^39^. For example, *Bacillus cereus* strains s as plant growth-promoting rhizobacteria have been used as biopesticides or biocontrol agents against various plant diseases^40–42^ and biofertilizers^43^. As previously reported *Bacillus* is an aerobic, rod-shaped, endospore-forming bacteria, and is a major community of the microbial flora in coastal ecosystems^44^. Moreover, several bacterial genera (*pseudomonads* and *bacilli*) have been founded as phosphate solubilizing bacteria their performance under in situ conditions is not reliable and therefore needs to be improved by using either genetically modified strains or co-inoculation techniques^45^. Plant growth and health-supporting bacteria of the *Bacillus* group due to their ability to form heat- and desiccation-resistant spores which can even provide a biological solution to the disease suppression of phytopathogenic fungi^46,47^. In this essence, slight changes in bacterial diversity and their changing forces could have a huge impact on plants and we analyze the effect of isolates on Maize, Canola, and Wheat.

Here we tried to have only one variable which is the texture of the soil. We tried to collect samples from locations with the same physiochemical identity. Our data show that the biomass of silt-base soil is higher than sandy soil. Interestingly, *pseudomonas* genera were absent in all of the samples from sand-based soil while *B. thuringiensis* have a substantial percentage of sand-based soil. The stress response of both sampling types shows no difference which might indicate none of the environments and soil textures did not induce chronic stress on the microbiome.

Last but not least our data suggest each silt or sand-based soil isolation influences plants differently and complexly which might have a better outcome in favor of the plants in their locational conditions. The result of the plant–microbe interaction test was expected as silt and sand-based soil show differences in biodiversity and biomass. Based on the heterogeneity of data on this matter more and more research and data should be available to have a better understanding of this complicated subject.

## Material and methods

### Soil sampling and determination of soil physical properties and synoptic data

Soil samples were taken from two coastal deserts in the north and south of Iran. Details of their geographic distribution and eco-physiological characterization were shown in Table 1. A total of 2 kg of soil samples were collected from 2 distinct sampling locations ranging in depth from 0 to 30 cm, and the samples were dried for 3 days at room temperature and in the dark before sifting. The soil samples were sieved using a 2 mm sieve to remove stones and other inert material before being stored in zip-top bags. Table 1 lists the soil samples' physical characteristics, including soil texture (sand 2–0.02 mm; silt 0.02–0.002 mm; clay 0.002 mm), pH, and the proportions of clay, silt, and sand. Synoptic data from the past 10 years (2009–2019), including the average annual temperature, maximum temperature, minimum temperature, average rainfall, average annual wind speed, and maximum wind speed, were obtained from the I.R.OF Iran Meteor (http://www.irimo.ir/far/index.php).

### Bacterial isolation and effect of manure-based medium on their growth

According to Chen et al. 2005, the soil-borne bacteria were isolated using direct-spreading method. For this essence soil samples were treated through a series of dilutions. The mixture of 1 g of soil sample was vortexed for 1 min after being suspended in 2 ml of sterile physiological saline (0.9% w/v NaCl). The mixture was then diluted serially (typically 10^–1^ to 10^–7^), and level 100 μl of the diluted soil samples were scattered on the surface of solidified plates using glass spreaders. The samples were then incubated for 1 to 3 days at 30 °C in an inverted posture without light. For bacterial isolation, we used eleven culture media including Nutrient Agar (NA), Nutrient Agar plus MnSO_4_ (NA + MnSO_4_), LB, Moller Hinton Agar (MHA), *Acidithiobacillus* (APH) medium, Violet Red Bile Lactose (VRB) agar medium, GYM *Streptomyces* medium, DPM medium, *Azospirillum* medium, *Azotobacter* medium and Manure based medium (MB).

To prepare MB medium, dry animal manure and distilled water (1:6 w/v) were combined to create MB medium, which was then let to sit at room temperature for 16 h. The resulting mixture was then centrifuged at 5000 rcf for 30 min after being filtered twice. The next stage involved adding Hoagland salts (10% w/v) to the final extract, adjusting the medium's pH to 5.8** ± **0.02, and autoclaving it for 20 min at 121 °C and 1.5 kPa. Before sterilization, bacteriological agar (1.5 w/v) was employed as a gelling agent to solidify the medium.

After bacterial isolation on NA, NA+ MnSO_4_, LB, MHA, APH, VRB, GYM, DPM, and Azospibrillum media, the growth of all isolates was evaluated on an MB medium. To investigate isolates biomass in the same condition, we elected MB medium. First, the bacteria were grown in the liquid form of NA, NA+ MnSO_4_, LB, MHA, APH, VRB, GYM, DPM, and *Azospirillum* and *Azotobacter* media at 30 °C for 48 h, then 10^3^ cells of each isolate were transferred to 48 wells plates containing MB medium, and plates were incubated at 30 °C for 10 h. Then, the growth of bacteria was read at an optical density (OD) of 630 nm 10 h after inoculation, the experiment was performed with three replicates. In the following step, CFU/ml equivalent to each OD was obtained by inoculating the uniform amount of liquid culture of the isolates on the solid form of MB medium at 30 °C for 16 h.

### Phenotypic characterization and biochemical identification of bacterial isolates

The morphological analysis of the cell shape, colony (i.e., shape, color, and size), and biochemical tests were used to identify the bacterial isolates. Biochemical characterization was carried out By using gram staining, KOH^27^, oxidase, and catalase tests. For this essence, following Bartholomew's method^28^, gram staining of bacteria was studied 48 h after inoculation on MHA, and the non-staining KOH method was used to confirm the results. Using 0.5 ml of a 10% hydrogen peroxide solution, a catalase test was conducted, and the generation of gas bubbles was monitored. Using biochemical oxidase discs, the oxidative activity of 27 isolates was investigated.

### Effect of abiotic stresses on bacterial isolates

To determine the effect of abiotic stresses on isolates alkaline (MH medium with pH  10), salinity (MH medium supplemented with the final concentration of 100 mM NaCl), osmotic [MH medium supplemented with 25% polyethylene glycol (PEG) Mn6000], and thermal stresses (MH medium incubated at 15 °C for cold stress and 60 °C for heat stress) were screened. For all experiments, the incubation period was 15 h, and plates were kept in a dark condition.

### MALDI-TOF MS identification of isolates

Soil bacterial isolates were subcultured twice on MHA and incubated at 30 °C for 24 h before MALDI-TOF MS measurement. Then ∼0.1 µg of cell material was directly transferred from a bacterial colony or smear of colonies to a MALDI target spot. After drying at laboratory temperature, sample spots were overlaid with 1 μl of matrix solution (10 mg/mL a-cyano-4-hydroxycinnamic acid in 50% acetonitrile and 2.5% trifluoroacetic acid) and each measurement was carried out in triplicate (technical replicates). MS analysis was performed on an Autoflex MALDI-TOF mass spectrometer (Bruker Daltonics, Germany) using Flex Control 3.4 software (Bruker Daltonics, Germany). Calibration was carried out with the use of the Bacterial Test Standard (Bruker Daltonics, Germany). Soil isolates with a valid MALDI-TOF MS score of 2 were undoubtedly assigned to the genus/species level. For bacterial classification and identification, BioTyper 3.1 software (Bruker Daltonics, Germany) equipped with MBT 6903 MPS Library (released in April 2016), the MALDI Biotyper Preprocessing Standard Method, and the MALDI Biotyper MSP Identification Standard Method adjusted by the manufacturer (Bruker Daltonics, Germany) were used. Only the highest score value of all mass spectra belonging to individual cultures (biological and technical replicates) was recorded^25^. The score between 2.3 and 3.00 shows highly probable species-level identification and between 2.0 and 2.29 represents genus-level identification and probable species level of identification. A score between 1.7 and 1.99 indicates probable genus-level identification^29^.

### Effects of bacterial isolates on plants growth

The Seed and Plant Improvement Institute of Karaj (Karaj, Iran; http://www.spii.ir/homepage.aspx?site=DouranPortal&tabid=1&lang=faIR) provided the maize, canola, and wheat seeds (Zea mays. Var Kosha; Brassica napus Var Nima; Triticum aestivum Var Kalate). In greenhouse trials, 2 × 10^3^ cells/seed of soil-borne isolates cultured in a manure-based medium were inoculated to maize, canola, and wheat plants. During the studies, sand that had been acid washed and autoclaved was used for planting. For three weeks, seedlings were kept under a 16/8 h day/night photoperiod with a 25 °C temperature. Three replications of a complete randomized block design were used for the colonization experiment's treatments. Under the bacterial treatments, measurements were made of the plant growth parameters including shoot dry biomass (mg), root dry biomass (mg), shoot length (cm), root length (cm), shoot density (mg/cm), root density (mg/cm), and shoot/root weight (mg). Samples were dried at 60 °C for three days to measure dry biomass.

### Statistical analysis

Statistical analysis was done by R software (version 4.1.3). One-way analysis of variance (ANOVA) was used to determine the significance of the experiment, and Fisher's protected Least Significant Difference (LSD) test with a P-value of 0.01 was performed to separate the means. Furthermore, PCA analysis has been carried out based on the Clustvis package and the SVD imputation approach.

### Ethics approval and consent to participate

All authors agree to the ethics and consent to participate in this article and declare that this submission follows the policies of *Scientific Reports*. Accordingly, the material is the author's original work, which has not been previously published elsewhere. The paper is not being considered for publication elsewhere. All authors have been personally and actively involved in substantial work leading to the paper and will take public responsibility for its content.

### Ethics for research involving plants

All authors confirmed that experimental research and field studies on plants, including receiving the seeds from the Seed and Plant Improvement Institute of Karaj, complied with relevant institutional, national, and international guidelines and legislation. Furthermore, methods were conducted according to the relevant guidelines and regulations.

## Data Availability

The datasets used and/or analyzed during the current study are available from the corresponding author upon reasonable request.
